# Cryptogenic Organizing Pneumonia with Sarcoidosis Overlap: An Atypical Case Study

**DOI:** 10.1155/2018/4316109

**Published:** 2018-06-20

**Authors:** Ajmal Nazir Neelambra, Vishak Acharya, Sowmya Sundararajan

**Affiliations:** ^1^Department of General Medicine, Kasturba Medical College, Mangalore, India; ^2^Department of Pulmonary Medicine, Kasturba Medical College, Mangalore, India; ^3^Kasturba Medical College, Mangalore, India

## Abstract

**Case Summary:**

We present a case of a young female with subacute symptoms of cough and progressive dyspnoea. On evaluation, the patient was diagnosed as cryptogenic organizing pneumonia based on her histopathological reports. However, her significant elevation of serum angiotensin-converting enzyme (SACE) levels which drop after treatment with oral steroids, relapse, and clinical presentation pointed towards sarcoidosis as clinical diagnosis.

**Discussion:**

Here, in this patient, transbronchial biopsy was suggestive of cryptogenic organizing pneumonia along with chest X-ray, and the HRCT finding was also favouring the same. But in this case, we have also seen elevated levels of serum ACE which dropped significantly to the normal level along with a complete clearance of lesions with systemic steroids, and this favours sarcoidosis. Also, the recurrence was in different areas of the lung, and lesions once again responded both clinically and radiologically to steroids with a consistent drop in serum angiotensin-converting enzyme (SACE) levels, which again is a feature common in sarcoidosis. In COP, often complete clearance of the lesions is seldom seen, even though they do respond to steroids but not as dramatically as in our case. Also, recurrence of the lesion with BOOP at different sites is uncommon as it generally progresses in the same site.

**Conclusion:**

This case report suggests that sarcoidosis as a possible cause of cryptogenic organizing pneumonia is worth considering with the mixed spectrum of presentation as in our case. And to our knowledge, this type of presentation of cryptogenic organizing pneumonia with sarcoidosis as an overlap disease is very rare, and this possibility needs to be explored by more series of such cases.

## 1. Introduction

Cryptogenic organizing pneumonia earlier known as bronchiolitis obliterans organizing pneumonia (BOOP) is clinically characterized by subacute or chronic respiratory disease for a duration of 2 weeks to 2 months. Usual presentation of cryptogenic organizing pneumonia will be persistent with dry cough and breathlessness. Pathologically, cryptogenic organizing pneumonia was defined by the presence of granulation tissue in the bronchiolar lumen, alveolar duct, and some alveoli, associated with a variable degree of interstitial and air space infiltration by mononuclear cells and foamy macrophages [1–3]. Various associations of cryptogenic organizing pneumonia with many drugs, connective tissue disorders, hematological malignancy, granulomatosis with polyangiitis, hypersensitivity pneumonitis, infections, inflammatory bowel disease, inhalation injury, irradiation injury, and transplantation have been reported in the literature, except for an unlikely cluster of patients with association of sarcoidosis and cryptogenic organizing pneumonia.

## 2. Case Summary

A 32-year-old female with no comorbid illness presented with dry cough and progressive dyspnoea for 3 weeks with no history of expectoration, fever, haemoptysis, night sweats, weight loss, myalgia, arthralgia, muscle weakness, or rash. Oral antibiotics were consumed with no symptomatic relief. She was a housewife with no history of exposure to mineral dust, silica, asbestos, fumes, and drugs.

Physical examination revealed a healthy well-built female with a BP of 100/80 mmHg, a respiratory rate of 22 breaths per minute, and a saturation of 94% in room air. No pallor, clubbing or cyanosis, lymphadenopathy, oedema, or skin lesions were observed.

Initial laboratory investigations showed an elevated ESR of 90 and an elevated serum angiotensin-converting enzyme (SACE) level of 83, as against a normal level of 52 IU, with normal hemogram and other biochemistry and electrolyte assays. Anti-nuclear antibody (ANA), anti-dsDNA, antineutrophil cytoplasmic antibody (C/P-ANCA), rheumatoid factor, and serology for HIV, HCV, and HBsAg were negative. Sputum for acid-fast bacilli and other routine bacteriology tests were negative.

**Figure 1:**
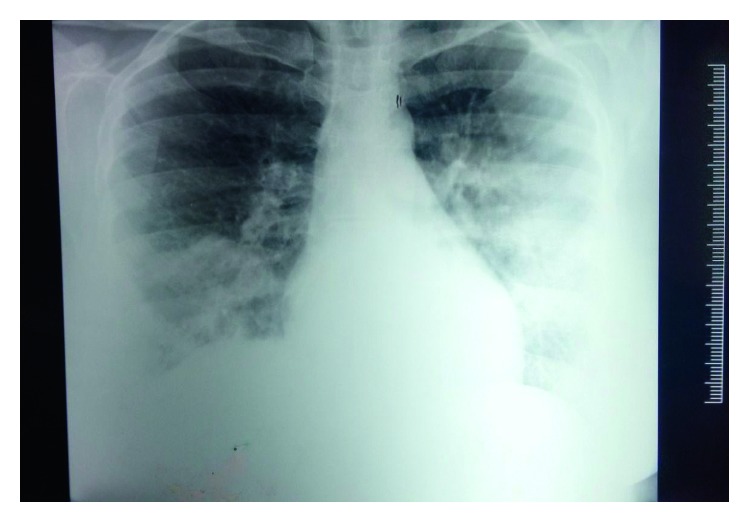
Chest X-ray showing bilateral interstitial infiltrates.

**Figure 2:**
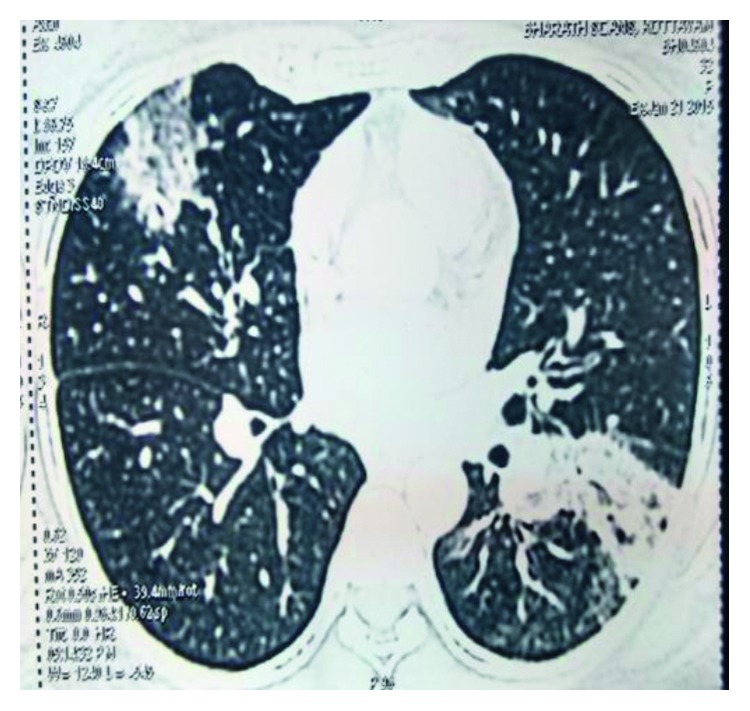


**Figure 3:**
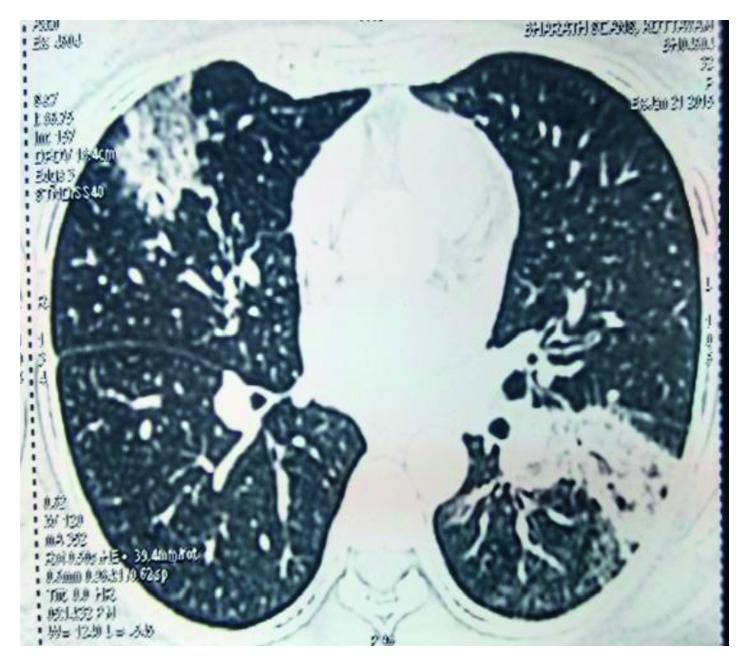
HRCT chest showing peripherally based patchy, subsegmental lesions of the upper lobe and left lower lobe.

**Figure 4:**
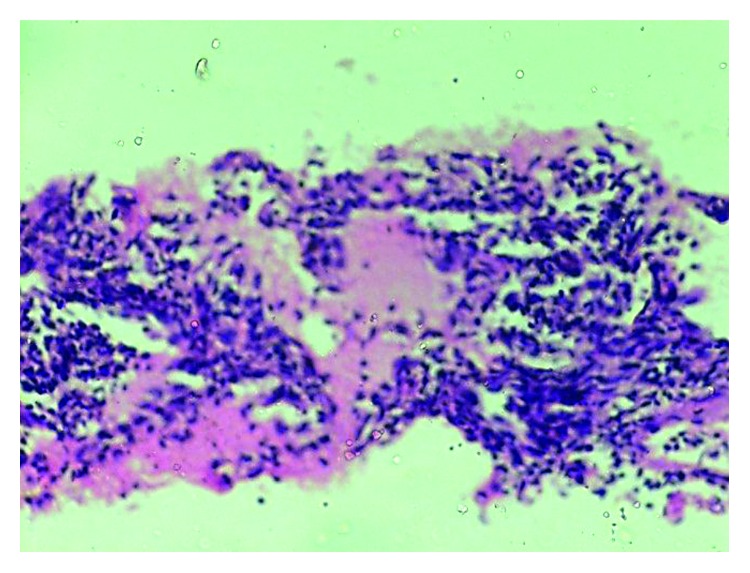
Low-power examination shows plugs of granulation tissue in bronchioles, alveolar ducts, and alveoli.

**Figure 5:**
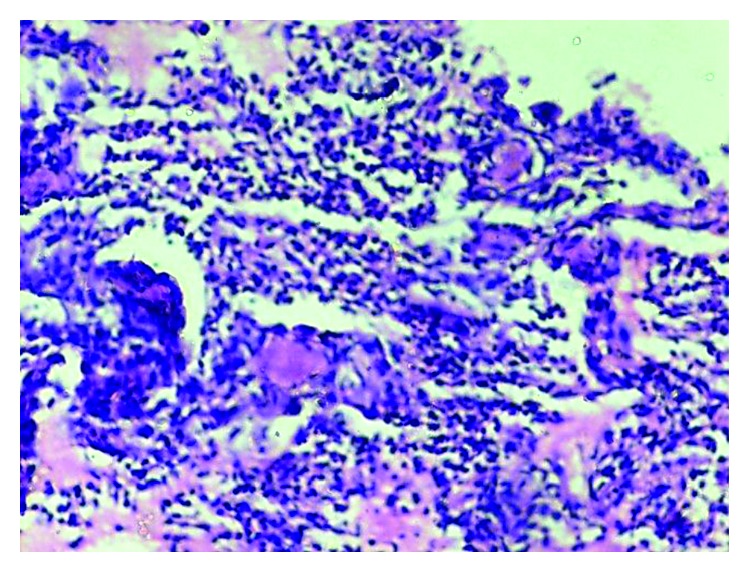
High-power examination shows Masson bodies that are rounded nodules of granulation tissue in alveolar spaces.

The pulmonary function test showed a mild restrictive pattern. Chest X-ray showed bilateral interstitial infiltrates (Figure 1). High-resolution computed tomography (HRCT) chest showed peripherally based patchy, subsegmental lesions of the upper lobe and left lower lobe (Figures 2 and 3). Bronchoscopy with brochoalveolar lavage (BAL) and transbronchial biopsies were done.BAL was found to be negative for malignancy. Cultures of bacterial pathogens and acid-fast bacilli were also found to be negative by GeneXpert. The BAL study showed endobronchial cells with alveolar dust-laden macrophages with acute and chronic inflammatory cells. Transbronchial lung biopsy was performed, and histology (Figures 4 and 5) showed air space consolidation with fibrinous exudation and polymorph and macrophage infiltration, along with polypoidal proteinous layer in the alveolar spaces composed of granulation tissue, with alveolar septa showing moderately dense chronic inflammatory infiltrates (Masson bodies), all were suggestive of cryptogenic organizing pneumonia (BOOP).

**Figure 6:**
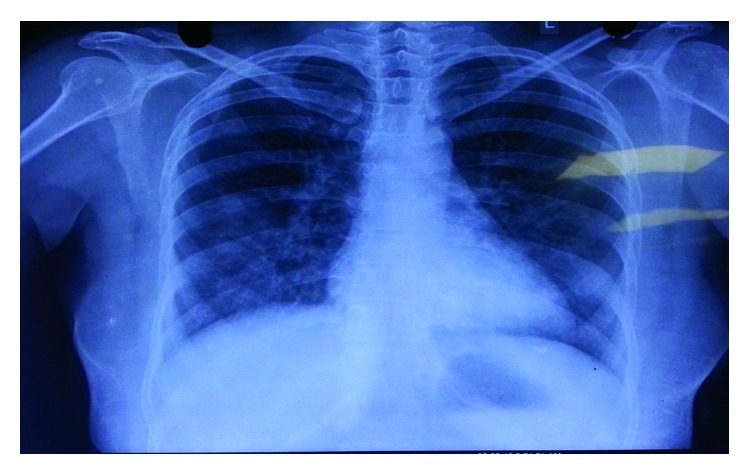
Chest X-ray showing clearance of bilateral interstitial infiltrates.

The patient was treated with systemic steroids to which the patient responded dramatically with a clinical and significant radiological clearance (Figure 6) and a decrease in serum ACE to normal. Steroid was gradually tapered and stopped after 3 months.

**Figure 7:**
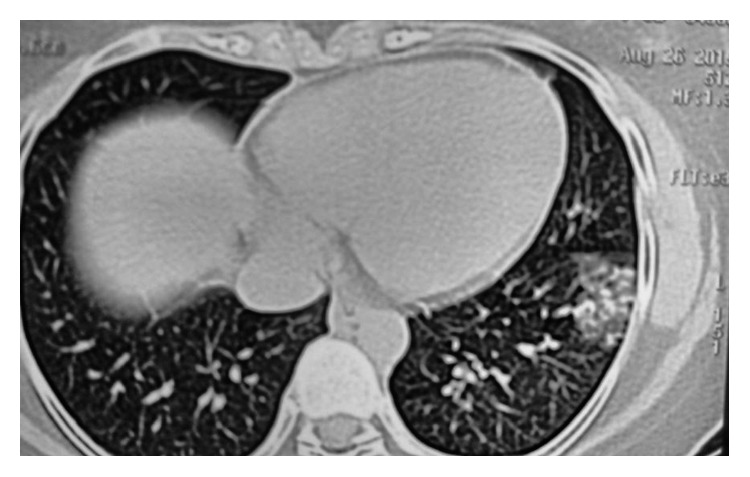
HRCT showing recurrence of bilateral patchy lesions in different zones.

The patient was asymptomatic for 5 months after withdrawal of steroids, following which her symptoms of cough and breathlessness reappeared. Chest radiograph and HRCT (Figure 7) showed recurrence of bilateral patchy lesions but in different zones of the lung. The patient was not willing for a repeat CT-guided FNAC and biopsy. The serum ACE levels again increased to pretreatment levels of 70 IU, and it again reached a nadir after another 3 months of a tapering course of steroids.

## 3. Discussion

The differential diagnosis of cryptogenic organizing pneumonia (BOOP) includes diseases like community-acquired pneumonia, idiopathic interstitial pneumonias, hypersensitivity pneumonitis, chronic eosinophilic pneumonia, and sarcoidosis [1, 4]. In our case, with the persistence of symptoms and lack of response to antibiotics, we diverged from bacterial or viral pneumonia. We excluded the diagnosis of idiopathic interstitial pneumonia with clinic-pathological collaboration. Cryptogenic organizing pneumonia (BOOP) has a similar clinical and radiographical presentation to subacute hypersensitivity pneumonitis. We excluded the disease because of the absence of a known exposure to an aetiological agent and the absence of poorly formed granulomas on lung biopsy. Chronic eosinophilic pneumonia can have a similar clinical and radiographical presentation to cryptogenic organizing pneumonia (BOOP). In chronic eosinophilic pneumonia, blood eosinophilia is usually higher than 1500/mm^3^ and bronchoalveolar eosinophilia is higher than 25%. In our case, blood and BAL eosinophils were within the normal range. Also in histopathological patterns, a significant eosinophilic infiltration is seen, and the eosinophilic infiltration is often accompanied by foci of necrosis and proteinaceous debris, termed eosinophilic microabscesses [1, 4]. With these findings, we excluded all other diseases and established the diagnosis of cryptogenic organizing pneumonia (BOOP).

In our case report, a 32-year-old female presented with dry cough and progressive dyspnoea with high ESR and elevated angiotensin-converting enzyme (SACE) levels, with the imaging study showing peripherally based patchy, subsegmental lesions of the upper lobe and left lower lobe and biopsy, suggestive of cryptogenic organizing pneumonia with granulation tissue and underwent remission of both clinical and radiological findings on treatment with systemic steroids.

Here, in this patient, transbronchial biopsy was suggestive of cryptogenic organizing pneumonia (BOOP) along with chest X-ray, and the HRCT finding was also favouring cryptogenic organizing pneumonia (BOOP). Here, we came to the final diagnosis of cryptogenic organizing pneumonia after excluding all other possible conditions as well.In the absence of other contributing disease processes, diagnosis of cryptogenic organizing pneumonia can be demonstrated by typical histological features in the patients with a compatible clinical and radiological pattern.

But in our case, we have also seen elevated levels of serum angiotensin-converting enzyme (SACE) which responded to oral steroids. The features that favoured sarcoidosis were that there was a significant clearance of lesions with steroids. Also, the recurrence was in different areas of the lung once steroids were discontinued, and lesions once again responded both clinically and radiologically to steroids with a consistent drop in serum angiotensin-converting enzyme (SACE) levels, suggesting a sarcoidosis aetiology to the disease.

In cryptogenic organizing pneumonia (BOOP), often complete clearance of the lesions is seldom seen, even though they do respond to steroids but not as dramatically as in our case. Also, recurrence of the lesion in cryptogenic organizing pneumonia (BOOP) at different sites is uncommon as it generally progresses in the same site. Distinguishing cryptogenic organizing pneumonia from sarcoidosis can be difficult. Cryptogenic organizing pneumonia frequently occurs in 40 to 60 years, and sarcoidosis usually occurs in the age group of 20 to 50 [5]. Both entities may present with almost same clinical symptoms. The histological appearance of cryptogenic organizing pneumonia and sarcoidosis differs as mononuclear cells and Langerhans giant-cell granulomas characterize acinar infiltrates of sarcoidosis, whereas cryptogenic organizing pneumonia is characterized by fibroblasts, myofibroblasts, and granulation buds in the distal airways and alveoli. The BAL study will show mononuclear cells in sarcoidosis and mixed cell populations with a modestly increased lymphocyte number in cryptogenic organizing pneumonia. Since our patient had a significant clinical, radiological, and pathological overlap of symptoms which could not be explained by either cryptogenic organizing pneumonia (BOOP) or sarcoidosis alone, an overlap of sarcoidosis-cryptogenic organizing pneumonia (BOOP) was a strong possibility.

## 4. Conclusion

In conclusion, for this patient, we came to a diagnosis of cryptogenic organizing pneumonia (BOOP) with sarcoidosis. Sarcoidosis as a possible cause of cryptogenic organizing pneumonia (BOOP) is worth considering with the mixed spectrum of presentation as in our case. And to our knowledge, this type of presentation of cryptogenic organizing pneumonia (BOOP) with sarcoidosis as an overlap disease is very rare, and this possibility needs to be explored by more series of such cases.
